# Supplementary material for: Supramolecular networks stabilise and functionalise black phosphorus
Source: Nat Commun. 2017 Nov 9;8:1385. doi: 10.1038/s41467-017-01797-6 (PMC5680224; doi:10.1038/s41467-017-01797-6)
Supplement: Supplementary file 1 — Supplementary Information [file 41467_2017_1797_MOESM1_ESM.pdf]

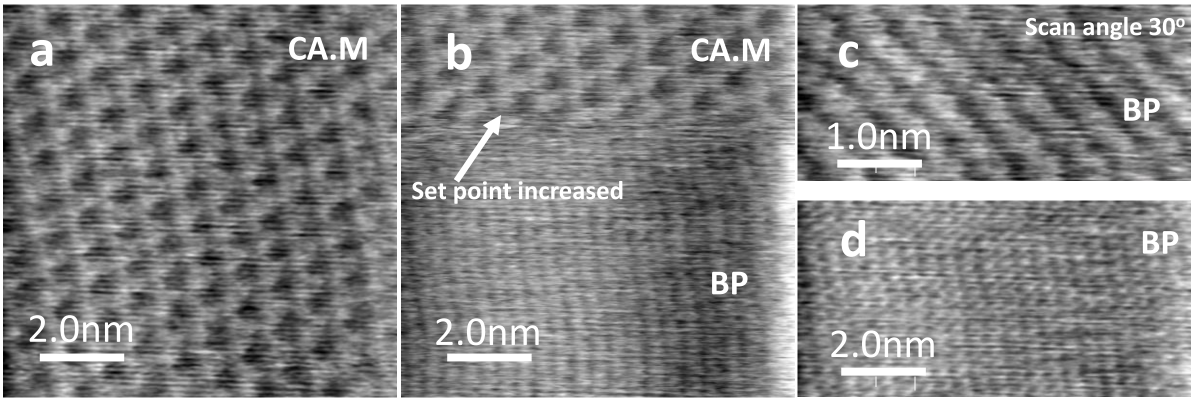

Supplementary Figure 1. Additional images of the terminated black phosphorus surface. AFM scan of CA.M/BP acquired in contact mode 1 week after the sample discussed in the main text was prepared. a – image of CA.M network; b – image of both CA.M network and underlying BP lattice acquired within a single scan by gradually increasing the imaging setpoint to remove the adsorbed molecular layer. c and d – additional contact mode scans of the BP surface showing rows of phosphorus atoms with scan c additionally showing intrarow resolution of pairs of phosphorus atoms.

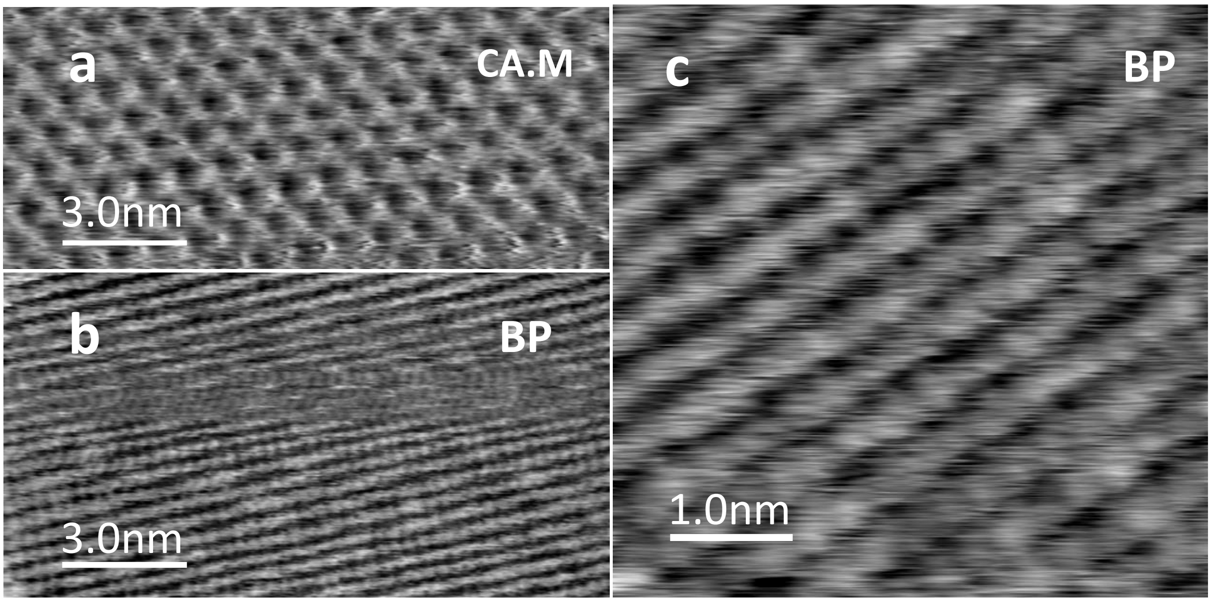

Supplementary Figure 2. Additional Images of the terminated black phosphorus surface. AFM scan of CA.M/BP acquired in tapping mode 3.5 weeks after the sample discussed in the main text was prepared; a – scan of CA.M network layer; b and c – scans of the underlying BP surface showing rows of phosphorus atoms.

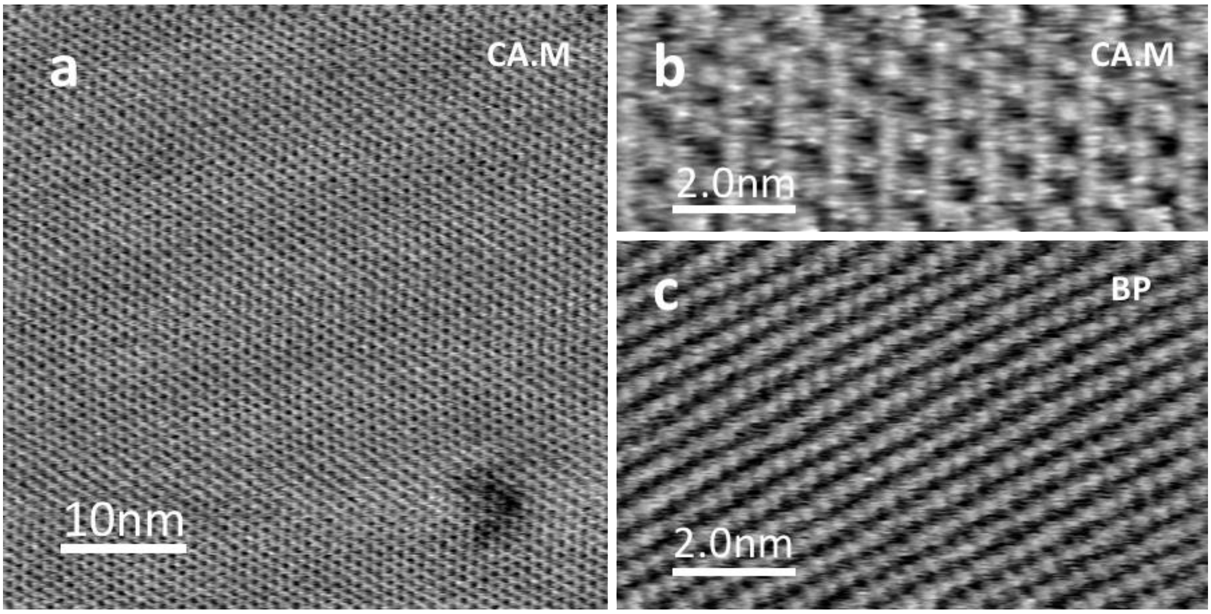

Supplementary Figure 3. Additional, larger area images of the terminated black phosphorus sample. AFM tapping mode scans of the CA.M/BP sample discussed in the main text acquired 1 month after the sample was prepare; a – scan of CA.M monolayer (the contrast has been reversed for clarity). b – a high resolution scan of CA.M showing single CA and M molecules; c – scan of BP after CA.M layer was removed with the tip. Zoomed areas of these regions are included in Figure 5 (main paper).

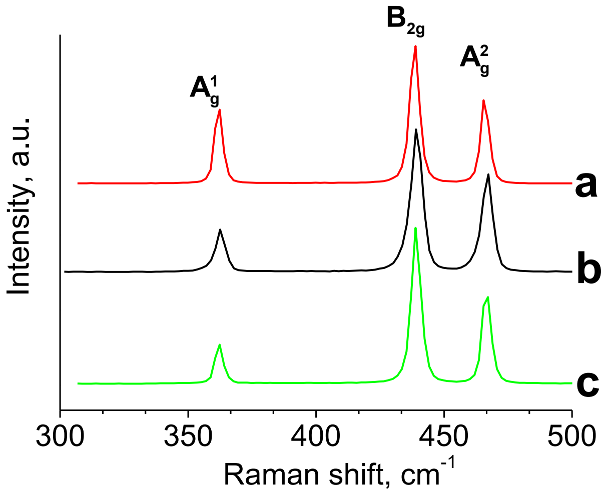

Supplementary Figure 4. Raman characterization of the terminated and unterminated black phosphorus surface. Raman spectra of exfoliated BP, a BP surface immediately after the deposition of CA.M, and the same sample one month later: a – freshly cleaved BP surface; b – 1 month old CA.M coated sample; c – freshly prepared CA.M sample. We observe peaks at 363 cm^{-1}, 438 cm^{-1} and 467 cm^{-1} which are assigned to the A^1_g, B^2_g and A^2_g modes respectively[2–7]. The intensity ratio of the A^1_g and A^2_g peaks was 0.95 for a freshly cleaved surface of BP crystal whereas the coated sample with CA.M network had a ratio of 0.41 immediately after formation; this ratio was unchanged after 1 month. These values are consistent with an oxide-free surface as suggested by Favron et al.[2]

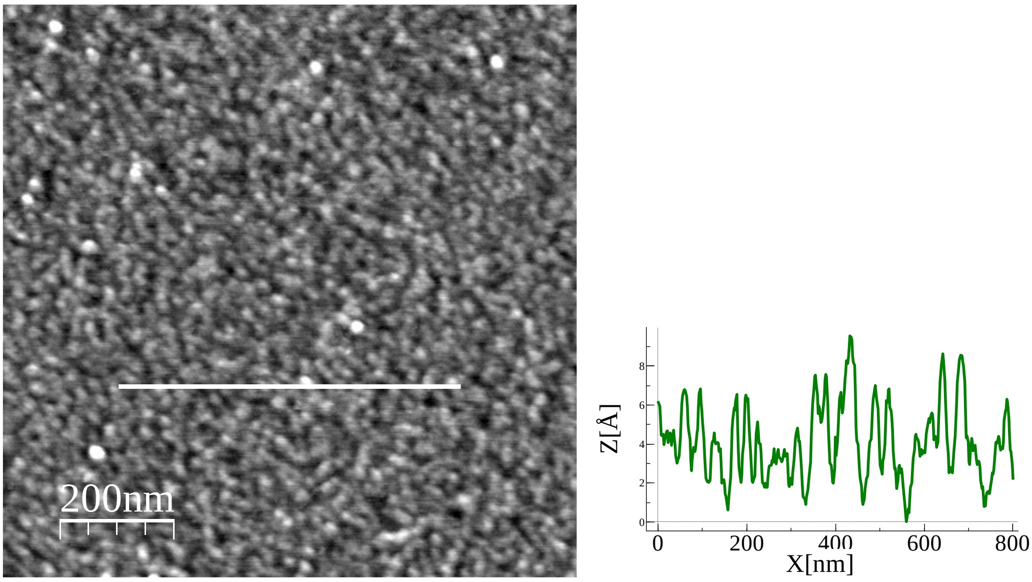

Supplementary Figure 5. Black phosphorus surface exposed to ethanol. The surface of BP after being washed with ~20ml of ethanol and dried in N_2-stream. A profile shows significantly increased roughness (~0.8nm) as compared to the profile shown on Figure 1 for a freshly cleaved surface of BP where surface is atomically flat.

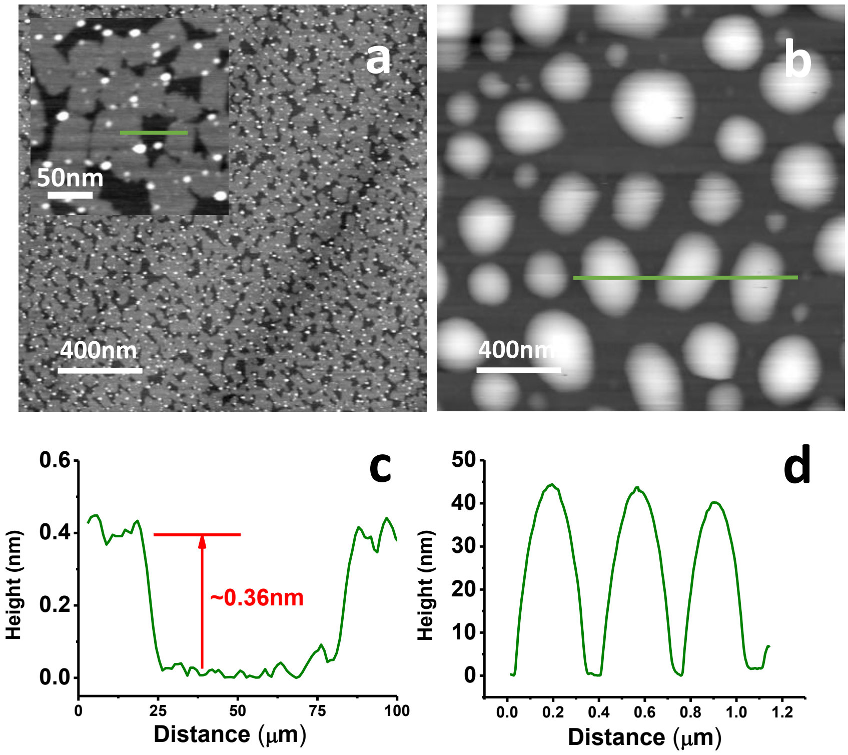

Supplementary Figure 6. The adsorption of 5,10,15,20-tetrakis(4-carboxylphenyl)porphyrin (TCPP) on the black phosphorus surface. AFM tapping mode scans and corresponding height profiles of BP surface with adsorbed 5,10,15,20-tetrakis(4-carboxylphenyl)porphyrin (TCPP) molecules: a – 2 μm x 2 μm scan showing surface coverage and an extra inset elaborating further on the shape of TCPP islands; b –the same area of the surface as shown in a, but imaged 16 hours later (the sample was left on the AFM scanner under ambient conditions). c – a profile showing step height of ~0.36nm for TCPP islands consistent with monolayer height islands. d – a profile showing large (~45nm) features on BP surface associated with atmospheric degradation. As observed for TMA on BP (main text), we observed that BP surface was stable, as judged by surface topography and integrity of molecular islands, for approximately 2-3 hours. After 12 hours the surface roughness had significantly increased with large protrusions (up to 45nm in height) associated with atmospheric degradation (scan b and profile d).

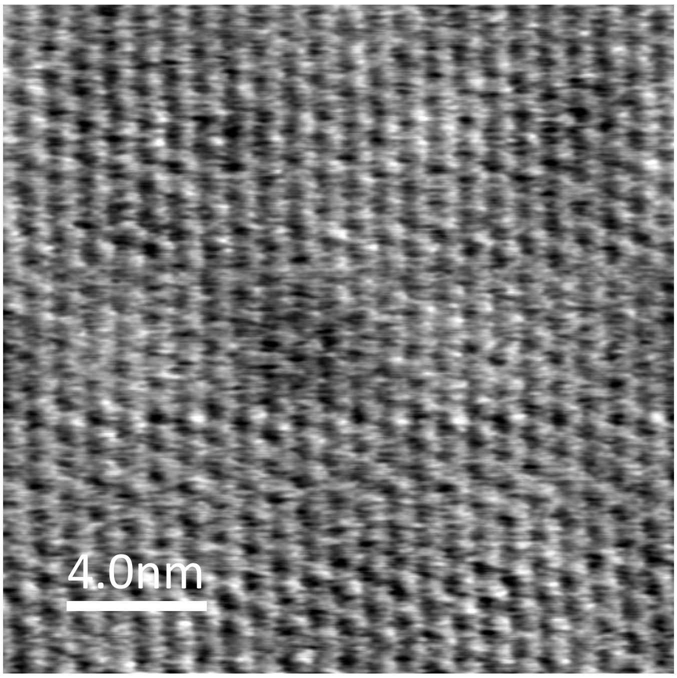

Supplementary Figure 7. A terminated black phosphorus sample three months after preparation. An AFM tapping mode scan of the CA.M network formed on BP acquired for a different sample to the one discussed in the main text. The images were acquired three months after the sample was prepared.

Supplementary References

1. Korolkov, V. V *et al.* van der Waals-Induced Chromatic Shifts in Hydrogen-Bonded Two-Dimensional Porphyrin Arrays on Boron Nitride. *ACS Nano* **9,** 10347–10355 (2015).
2. Favron, A. *et al.* Photooxidation and quantum confinement effects in exfoliated black phosphorus. *Nat. Mater.* **14,** 826–832 (2015).
3. Erande, M. B., Suryawanshi, S. R., More, M. A. & Late, D. J. Electrochemically Exfoliated Black Phosphorus Nanosheets - Prospective Field Emitters. *Eur. J. Inorg. Chem.* **2015,** 3102–3107 (2015).
4. Al-Masoodi, A. H. H., Ahmed, M. H. M., Latiff, A. A., Arof, H. & Harun, S. W. Q-Switched Ytterbium-Doped Fiber Laser Using Black Phosphorus as Saturable Absorber. *Chinese Phys. Lett.* **33,** 54206 (2016).
5. Tayari, V. *et al.* Two-dimensional magnetotransport in a black phosphorus naked quantum well. *Nat. Commun.* **6,** 7702 (2015).
6. Liu, X. K. *et al.* Black Phosphorus Based Field Effect Transistors with Simultaneously Achieved Near Ideal Subthreshold Swing and High Hole Mobility at Room Temperature. *Sci. Rep.* **6,** 8 (2016).
7. Castellanos-Gomez, A. *et al.* Isolation and characterization of few-layer black phosphorus. *2D Mater.* **1,** 25001 (2014).
